# The true natural cycle frozen embryo transfer - impact of patient and follicular phase characteristics on serum progesterone levels one day prior to warmed blastocyst transfer

**DOI:** 10.1186/s12958-023-01136-z

**Published:** 2023-09-18

**Authors:** Sezcan Mumusoglu, Murat Erden, Irem Yarali Ozbek, Onur Ince, Sandro C. Esteves, Peter Humaidan, Hakan Yarali

**Affiliations:** 1 Department of Obstetrics and Gynecology, Hacettepe University School of Medicine, Ankara, Turkey; 2 Anatolia IVF and Women Health Centre, Ankara, Turkey; 3 Department of Obstetrics and Gynecology, Kutahya Health Sciences University, Kutahya, Turkey; 4 Faculty of Arts and Science, Department of Statistics, Middle East Technical University, Ankara, Turkey; 5 Androfert, Andrology, and Human Reproduction Clinic, Referral Center for Male Reproduction, Campinas, SP Brazil; 6 Department of Clinical Medicine, Aarhus University, Aarhus, Denmark; 7 The Fertility Clinic, Skive Regional Hospital, Resenvej 25, Skive, Denmark

**Keywords:** Natural cycle, Frozen embryo transfer, Follicular phase, Luteinized unruptured follicle, Luteal phase support, Progesterone

## Abstract

**Background:**

In a true-natural cycle (t-NC), optimal progesterone (P_4_) output from the corpus luteum is crucial for establishing and maintaining an intrauterine pregnancy. In a previous retrospective study, low P_4_ levels (< 10 ng/mL) measured one day before warmed blastocyst transfer in t-NC were associated with significantly lower live-birth rates. In the current study, we aim to examine the relationship between patient, follicular-phase endocrine and ultrasonographic characteristics, and serum P_4_ levels one day prior to warmed blastocyst transfer in t-NC.

**Method:**

178 consecutive women undergoing their first t-NC frozen embryo transfer (FET) between July 2017-August 2022 were included. Following serial ultrasonographic and endocrine monitoring, ovulation was documented by follicular collapse. Luteinized unruptured follicle (LUF) was diagnosed when there was no follicular collapse despite luteinizing-hormone surge (> 17 IU/L) and increased serum P_4_ (> 1.5 ng/mL). FET was scheduled on follicular collapse + 5 or LH surge + 6 in LUF cycles. Primary outcome was serum P_4_ on FET − 1.

**Results:**

Among the 178 patients, 86% (n = 153) experienced follicular collapse, while 14% (n = 25) had LUF. On FET-1, the median serum luteal P_4_ level was 12.9 ng/mL (IQR: 9.3–17.2), ranging from 1.8 to 34.4 ng/mL. Linear stepwise regression revealed a negative correlation between body mass index (BMI) and LUF, and a positive correlation between follicular phase peak-E_2_ and peak-P_4_ levels with P_4_ levels on FET-1. The ROC curve analyses to predict < 9.3 ng/mL (< 25th percentile) P_4_ levels on FET-1 day showed AUC of 0.70 (95%CI 0.61–0.79) for BMI (cut-off: 23.85 kg/m^2^), 0.71 (95%CI 0.61–0.80) for follicular phase peak-P_4_ levels (cut-off: 0.87 ng/mL), and 0.68 (95%CI 0.59–0.77) for follicular phase peak-E_2_ levels (cut-off: 290.5 pg/mL). Combining all four independent parameters yielded an AUC of 0.80 (95%CI 0.72–0.88). The adjusted-odds ratio for having < 9.3 ng/mL P_4_ levels on FET-1 day for patients with LUF compared to those with follicle collapse was 4.97 (95%CI 1.66–14.94).

**Conclusion:**

The BMI, LUF, peak-E_2,_ and peak-P_4_ levels are independent predictors of low serum P_4_ levels on FET-1 (< 25th percentile; <9.3 ng/ml) in t-NC FET cycles. Recognition of risk factors for low serum P_4_ on FET-1 may permit a personalized approach for LPS in t-NC FET to maximize reproductive outcomes.

## Introduction

Efficient and safe embryo vitrification techniques have contributed to a marked increase in frozen embryo transfer (FET) cycles worldwide during the last decade [1, 2]. Currently, low-quality evidence suggests that the hormone replacement treatment (HRT) protocol is associated with lower live birth rates (LBRs) compared to the natural cycle (NC) FET [3, 4]. Moreover, the NC seems to be associated with more favorable maternal, obstetric, and perinatal outcomes compared to the HRT protocol [5]. After adjusting for potential confounders, hypertensive disorders of pregnancy, including pre-eclampsia, significantly increase following an HRT cycle compared to NC due to the absence of a corpus luteum [5–7]. Furthermore, the incidence of very preterm birth and preterm birth, postpartum hemorrhage, and cesarean section significantly rise after HRT when compared to NC [5–7]. Therefore, recently a “back to nature” approach, which advocates an expanded use of NC FET, was suggested by some authors [8, 9].

In a true NC (t-NC) FET, the day of ovulation should be precisely identified, following serial endocrine and transvaginal ultrasonographic monitoring to schedule blastocyst transfer. A fundamental question is whether the mid-luteal serum progesterone (P_4_) levels impact reproductive outcomes in a t-NC FET. In a t-NC, an optimal P_4_ output from the corpus luteum, originating from the mono-follicular development, is crucial for establishing and maintaining an intrauterine pregnancy [10]. In a previous retrospective study, low serum P_4_ levels (< 10 ng/mL) measured one day before warmed blastocyst transfer were associated with significantly lower LBRs [11]. However, the pulsatile secretion of P_4_ during the mid-luteal phase is challenging for serum P_4_ monitoring in t-NC [12]. Until now, the most common practice has been to perform t-NC FET without mid-luteal serum P_4_ monitoring, but instead administering routine exogenous luteal phase support (LPS) to overcome possible luteal phase defects in a subset of natural cycles. However, three randomized controlled trials (RCTs) reported conflicting results on reproductive outcomes following LPS administration in t-NC [13–15].

Given that in medicine “one treatment does not fit all”, the current study sought to explore the patient, endocrine, as well as ultrasonographic characteristics that could identify those women who are at risk of having low serum P_4_ levels one day prior to warmed blastocyst transfer employing t-NC.

## Materials and methods

### Design and study population

A cohort study of 187 consecutive ovulatory women who underwent their first t-NC warmed blastocyst transfer cycle at Anatolia IVF and Women’s Health Center, Ankara, Turkey, from July 2017 to August 2022.

The inclusion criteria for the study were as follows: (i) female age ≤ 45 years old; (ii) patients with regular menstrual cycles and living in the town to permit frequent endocrine and ultrasonographic monitoring; (iii) available serum P_4_ levels one day prior to warmed blastocyst transfer (FET-1).

Following the inclusion criteria, a total of nine cycles were excluded: five due to lack of follicular growth, two due to vaginal bleeding, and one due to the patient’s request to postpone the FET. Thus, a total of 178 cycles were included in the final analysis. Due to timely and frequent endocrine and ultrasonographic monitoring, no patient had ovulation prior to starting monitoring.

The Institutional Review Board of Hacettepe University approved the study protocol (Protocol number: KA-21,116).

### t-NC protocol

Transvaginal ultrasonography was performed on day 2 or 3 of menses to rule out any cyst or corpus luteum prevailing from the previous cycle. If t-NC was performed immediately after a failed fresh transfer or a freeze-all cycle with a persistent corpus luteum, cycle cancellation was undertaken in cycles with serum P_4_ > 1.5 ng/mL on day 2 or 3 of menses. Transvaginal ultrasonographic monitoring started on days 8–10. When the leading follicle attained a mean diameter of 14–15 mm, daily transvaginal ultrasonographic and endocrine monitoring (E_2_, LH, and P_4_ measurements) was performed. The day of ovulation was documented by follicular collapse as defined by the complete disappearance of the follicle or reduction in volume with thickening of the follicle wall [16]. Warmed blastocyst transfer was scheduled five days after follicular collapse [17]. A diagnosis of luteinized unruptured follicle (LUF) was made when there was no follicular collapse despite a documented onset of the LH surge (> 17 IU/L) [18] and an increased serum P_4_ level (> 1.5 ng/ml) one or two days after the onset of the LH surge. Follicular collapse was not noted in such cases despite two to three daily ultrasonographic monitoring following serum P_4_ increase (> 1.5 ng/ml). In LUF cycles, the day of warmed blastocyst transfer was scheduled for the onset of the LH surge + 6 day. All cycles included were t-NC, thus no human chorionic gonadotropin (hCG) was used for trigger and no LPS was administered. Luteal serum P_4_ levels were monitored on FET-1.

Two different policies were adopted in the execution of t-NC FET during July 2017-August 2022. Thus, during July 2017-June 2020, serum P_4_ levels on FET − 1 were routinely monitored (n = 84), and warmed blastocyst transfer was canceled when serum P_4_ levels were lower than an arbitrary cut-off point (< 7 ng/mL) (n = 7), and no LPS was administered for those patients ≥7 ng/mL. During July 2020-August 2022 (n = 94), in addition to canceling warmed blastocyst transfer in patients with serum P_4_ levels < 7 ng/mL (n = 6), a daily subcutaneous (s.c.) rescue progesterone administration strategy was adopted for patients with serum P_4_ levels between 7 and 10 ng/mL (n = 18). Cancellation of those cycles with serum P_4_ < 7 ng/mL and employment of a rescue progesterone administration for serum P_4_ levels 7–10 ng/mL in the latter period did not permit us to evaluate the impact of serum P_4_ on reproductive outcomes in the current study.

### Laboratory procedures

Serum P_4_ and E_2_ were measured using the commercially available VIDAS® ImmunoDiagnostic Assay System as an automated quantitative enzyme-linked fluorescent assay (bioMérieux, Marcy l’Etoile, France). The assay sensitivity was 0.25 ng/mL for serum P_4_ and 9 pg/mL for serum E_2_. The intra-assay coefficient of variations was 3.97–14.30% and 2.2–7.5%, and the inter-assay coefficient of variations was 3.10–24.30% and 3.2–9.5% for serum P_4_ and E_2_, respectively. All serum P_4_ measurements one day prior to warmed blastocyst transfer were performed at 12.00–1.00 pm.

Serum LH was measured using the Cobas e 601 analyzers, employing the Elecsys LH immunoassay (Roche Diagnostics International Ltd, Rotkreuz, Switzerland). The assay uses a sandwich test principle and a measuring range of 0.100–200 IU/L, as defined by the lower detection limit and the maximum of the master curve. The coefficients of variation for repeatability and intermediate precision were 0.6–1.2% and 1.6–2.2%, respectively.

### Outcome measures

The primary outcome measure was the serum P_4_ level on FET − 1. The follicular phase was defined as the period starting from the first day of active vaginal bleeding until ovulation. Follicular phase peak-E_2_, LH, and P_4_ levels denoted the maximum levels attained during the late follicular phase. The area under the curve (AUC) of serum E_2_, LH, and P_4_ was calculated. Ongoing pregnancy rate is defined as a gestational sac with fetal cardiac activity greater than 12 weeks of gestation.

### Statistical analyses

Statistical Package for the Social Sciences Version 23.0 (IBM Corp., Armonk, NY, USA), R Version 3.6.1 (https://www.r-project.org/) and Minitab 21.1.1 Statistical Software (Minitab, State College, PA) were used for data analysis. Distribution characteristics of variables were visually assessed using histograms, box plots, and Q-Q plots and analyzed using Kolmogorov–Smirnov, and Shapiro–Wilk tests. Continuous variables with normal distribution were expressed as mean ± SD, whereas median [interquartile range (IQR); 25th and 75th percentiles] with the non-Gaussian distribution. Chi-squared and Fisher’s exact tests were used to compare the categorical variables. Pearson and Spearman’s correlations were used to test the correlation between cycle characteristics and serum P_4_ levels one day before warmed blastocyst transfer. Two-tailed p-value < 0.05 was considered statistically significant.

To identify the independent predictors of serum P_4_ levels on FET-1, the linear stepwise regression model was performed. The initial model included age, body mass index (BMI), antral follicle count (AFC), follicular phase length, follicle diameter one day prior to ovulation, endometrial thickness one day prior to ovulation, follicular phase peak-E_2_, peak-LH, peak-P_4_ levels, and LUF as covariates; the included variables in the model did not show a strong correlation (correlation coefficients < 0.60). To determine the most relevant variables, a stepwise elimination approach was performed with entry and removal significance levels set at α = 0.10 and α = 0.15, respectively. The normality of residuals was assessed using the Shapiro-Wilk test, while heteroscedasticity was checked using the studentized Breusch-Pagan test. To evaluate linearity, second-degree polynomials of the variables were included in the initial model, and a Box-Cox transformation was performed. Among the different transformations tested, the square root transformation (λ = 0.464, rounded to 0.5) exhibited a linear relationship with the predictor variables. This transformation satisfied the assumptions of homoscedasticity and normality of residuals. The effect size was presented as β-Coefficient [95% Confidence Interval (CI)].

Receiver Operator Characteristics (ROC) curve analysis was performed, using the coefficients derived from the generalized linear stepwise regression model to assess the significance of each parameter or combination of parameters in predicting low serum P_4_ level on FET-1(< 25th percentile). The area under the curve (AUC; 95% CI) was calculated using the ROC curves and the Youden index was used to identify the cut-off of BMI, follicular phase peak-E_2_, and peak-P_4_ levels associated with low serum P_4_ level on FET-1. A multivariate logistic regression model was conducted to identify the odds ratio (OR) of having low serum P_4_ level on FET-1 (< 25th percentile) in patients with LUF compared to those with follicle collapse.

## Results

### Patient demographics and follicular phase characteristics

Patient demographics, embryological data and cycle characteristics of the 178 t-NC cycles are shown in Table 1. The median age was 36 years (IQR: 32–40), BMI was 23.1 kg/m^2^ (IQR: 21.1–25.9), AFC on day 2/3 was 14 (IQR: 9–18), and the follicular phase length was 13 days (IQR: 11–15). The median follicle diameter one day prior to ovulation was 19.2 mm (IQR: 17.8–20.9). The mean$\pm$SD endometrial thickness one day prior to ovulation was 10.4$\pm$2.0 mm. Follicular collapse was observed in 153 patients (86%), whereas the remaining 25 patients (14%) experienced LUF.

**Table 1:** Patient demographics at baseline, embryological data, and true natural cycle characteristics

Age, years	36 (32–40)
Body mass index, kg/m^2^	23.1 (21.1–25.9)
Antral follicle count on Day 2/3, n	14 (9–18)
Cause of infertility, n (%)	
Unexplained infertility	57 (32.0)
Male factor	54 (30.3)
Advanced maternal age and/or diminished ovarian reserve	48 (27.0)
Tubal factor	6 (3.4)
Endometriosis	6 (3.4)
Monogenic disorders	7 (3.9)
Duration of infertility, months	28 (15.75–48.0)
Number of previous IVF cycles, median (minimum-maximum)	0 (0–8)
Previous childbirth, n (%)	35 (19.7)
Day of vitrification, n (%)	
Day 5	118 (66.3)
Day 6	60 (33.7)
Blastocyst morphology^a^, n (%)	
Excellent	20/165 (12.1)
Good	74/165 (44.9)
Average	66/165 (40.0)
Poor	5/165 (3.0)
Number of patients with PGT-A, n (%)	57 (32.0)
Number of patients with PGT-M, n (%)	7 (3.9)
Number of blastocyt(s) transferred	1 (1–2)
Number of cycles with single blastocyst transfer, n (%)	130/165 (78.8)
Follicular phase length, day	13 (11–15)
Follicle diameter one day prior to ovulation, mm	19.2 (17.8–20.9)
Endometrial thickness one day prior to ovulation, mm	10.4 ± 2.0
Number of patients with follicular collapse, n (%)	153 (86)
Number of patients with luteinized unruptured follicle ^b^, n (%)	25 (14)

Values are given as mean ± SD, median (25th – 75th percentiles), or n (%)

IVF: in-vitro fertilization, PGT-A: preimplantation genetic testing for aneuploidy, PGT-M: preimplantation genetic testing for monogenic disorders

^a^ Blastocyst grading was categorized as excellent (3AA, 4AA, 5AA), good (3,4,5,6 AB or BA), average (3,4,5,6 BB or AC or CA), and poor (3,4,5,6 BC or CC). When more than one embryo was transferred, the one with the best morphological grading was included in the analysis

^b^ Luteinized unruptured follicle (LUF) was diagnosed when there was no follicular collapse despite an LH surge (> 17 IU/L) and increased serum P_4_ (> 1.5 ng/mL)

Since the duration of the follicular phase differed among the study population (range from 8 to 21 days), 12 patients had only one day, 34 patients had two days and the remaining 132 had three days of endocrine and ultrasonographic monitoring before ovulation (Table 2). The median serum LH level displayed a ~ 2-fold increase from ovulation − 3 day to the ovulation − 2 day [19.4 IU/L, (IQR: 13.9–26.2) versus 9.8 IU/L (IQR: 8.2–13.1), respectively], and reached its peak on ovulation − 1 [41.3 IU/L (IQR: 30.1–56.0)]. The median serum E_2_ levels peaked on the ovulation − 2 day at 301.0 pg/mL (IQR: 236.0–364.5). The median serum P_4_ levels peaked on the ovulation − 1 day at 1.0 ng/mL (IQR: 0.8–1.2). On FET-1, the median serum P_4_ level was 12.9 ng/mL (IQR: 9.3–17.2 ng/mL), with a range of 1.8 ng/mL to 34.4 ng/mL. The median serum P_4_ concentration on FET-1 was significantly lower in patients with LUF when compared to those with follicle collapse 9.3 ng/mL (IQR: 5.5–15.4) versus 13.6 ng/mL (IQR: 10.3–17.3), respectively, p = 0.002)].

**Table 2:** The daily endocrine and ultrasonographic monitoring data as categorized according to the day of ovulation

	Ovulation − 3 days n = 132	Ovulation − 2 days n = 166	Ovulation − 1 day n = 178	Ovulation + 4 days (FET − 1 day) n = 178
LH level, IU/L	9.8 (8.2–13.1)	19.4 (13.9–26.2)	41.3 (30.1–56.0)	NA
E_2_ level, pg/mL	223.0 (168.5–272.5)	301.0 (236.0–364.5)	235.0 (170.8–298.5)	NA
P_4_ level, ng/mL	0.5 (0.3–0.7)	0.6 (0.4–0.9)	1.0 (0.8–1.2)	12.9 (9.3–17.2)
Follicle diameter, mm	16.3 (15.2–17.7)	18.0 (16.8–19.5)	19.2 (17.8–20.9)	NA

Data are presented as median (25th – 75th percentiles). LH, luteinizing hormone; E_2_, estradiol; P_4_, progesterone; FET, frozen embryo transfer; NA, not available

Of the study population undergoing blastocyst transfer, the overall ongoing pregnancy rate was 56.4% (93 out of 165). The multiple pregnancy rate per ongoing pregnancy was 7.5% (7/93).

### Covariates affecting serum P_4_ concentrations on FET − 1

#### Univariate analysis

The correlation between age, BMI, AFC, follicular phase endocrine/ultrasonographic parameters, and serum P_4_ levels on FET − 1 are given in Table 3. There were significant positive correlations between serum P_4_ levels on FET-1 and follicle diameter one day prior to ovulation (*r* = 0.235, p = 0.002), AUC-E_2_ level (*r* = 0.406, p < 0.001), the follicular phase peak-E_2_ level (*r* = 0.480, p < 0.001), the follicular phase AUC-P_4_ level (*r* = 0.286, p < 0.001), and the follicular phase peak-P_4_ level (*r* = 0.351, p < 0.001). In contrast, negative correlations were seen between BMI (*r*=-0.378, *P* < 0.001), LUF (*r*=-0.236, p = 0.002), and serum P_4_ levels on FET − 1.

**Table 3:** Univariate correlation between female demographics, follicular phase characteristics, and serum P_4_ levels one day prior to warmed blastocyst transfer (FET-1)

Demographic and follicular phase characteristics	*Correlation (r* ^*a*^ *)*	*P*-value
Age, years	-0.078	0.30
Body mass index, kg/m^2^	-0.378	**< 0.001**
Antral follicle count, n	0.032	0.67
Follicular phase length, days	0.079	0.31
Follicle diameter one day prior to ovulation, mm	0.235	**0.002**
AUC-E_2_ level, pg/mL	0.406	**< 0.001**
Peak E_2_ level, pg/mL	0.480	**< 0.001**
AUC-LH level, IU/L	0.072	0.36
Peak LH level, IU/L	-0.006	0.94
Follicular phase AUC-P_4_ level, ng/mL	0.286	**< 0.001**
Follicular phase peak P_4_ level, ng/mL	0.351	**< 0.001**
Endometrial thickness one day prior to ovulation, mm ^b^	-0.07	0.33
Luteinized unruptured follicle ^c^	-0.236	**0.002**

^a^ Spearman correlation test.; ^b^ Pearson correlation test; ^c^ A point-biserial correlation

AUC: area under the curve

To delineate the impact of patient demographics and endocrine and ultrasonographic characteristics on serum P_4_ levels on FET-1, comparisons were made between the < 10th, 10–24th, 25–49th, 50–90th, and > 90th percentiles (Table 4). The thresholds of serum P_4_ levels on FET-1 for the 10th and 25th percentiles were 6.81 ng/ml and 9.30 ng/ml, respectively. When the < 10th and 10–25th serum P_4_ percentile groups were compared with those of 25–49th, 50–90th, and > 90th, the following significant differences were noted: the median BMI was significantly higher in the < 10th and 10–25th percentile groups compared to those of the 50–90th and > 90th percentiles. The median follicle diameter one day prior to ovulation was significantly lower in the < 10th and 10–25th groups compared to that of the > 90th percentile group. The median follicular phase peak-E_2_ level was significantly lower in the < 10th and 10–25th percentile groups compared to those of the 50–90th and > 90th percentiles. Finally, the median follicular phase peak-P_4_ level was significantly lower in the < 10th and 10–25th percentile groups compared to those of the 25–49th, 50–90th, and > 90th percentiles. Of the 17 patients in the < 10th percentile group, a total of 8 patients (47%) had LUF; this rate was significantly higher than those noted in the 10–25th, 25–49th, 50–90th, and > 90th percentile groups (Table 4).

**Table 4:** Comparison of the baseline demographic features and true natural cycle characteristics of patients at different serum P_4_ percentiles on FET-1

Characteristics	< 10p (n = 17) < 6.81 ng/ml	10–24p (n = 26) 6.81–9.30 ng/ml	25–49p (n = 46) 9.31–12.94 ng/ml	50–90p (n = 72) 12.95–21.72 ng/ml	> 90p (n = 17) > 21.72 ng/ml	P*
Female age, years	38.0 (33.0–40.0)	37.0 (33.0–39.0)	35.5 (32.0–39.0)	34.5 (31.5–40.0)	38.0 (31.0–39.0)	0.870
Body mass index, kg/m^2^	25.7 (22.2–29.7)^a^	25.3 (22.3–27.6)^a^	23.2 (21.2–26.9)	22.4 (20.8–24.8)	21.2 (20.1–23.7)	**0.001**
Antral follicle count, n	15 (7.0–21.0)	13 (8.0–18.5)	15 (8.0–18.0)	15 (9.0–19.0)	17 (9.5–18.5)	0.809
Follicular phase length, days	13.0 (10.5–15.5)	13.5 (11.0–14.3)	12.0 (10.8–14.0)	13.0 (11.0–15.0)	13.0 (12.5–15.5)	0.202
Follicle diameter one day prior to ovulation, mm	18.8 (17.5–21.4)^b^	18.6 (17.2–20.6)^b^	18.8 (17.6–20.4)^b^	19.4 (18.1–21.0)^b^	20.6 (19.0–22.6)	**0.012**
Peak-E_2_ level, pg/mL	275.5 (198.0–335.2)^a^	260.0 (209.5–323.5)^a^	276.0 (196.2–334.2)^a^	350.0 (292.5–413.0)	393.0 (330.2–488.5)	**< 0.001**
Peak-LH level, IU/L	40.4 (36.1–53.3)	44.4 (31.7–65.4)	44.9 (35.2–57.9)	43.6 (30.8–57.7)	45.7 (37.1–55.3)	0.993
Follicular phase peak-P_4_ level, ng/mL	0.8 (0.6–1.0)^c^	0.8 (0.6–0.9)^c^	0.9 (0.7–1.2)	1.0 (0.9–1.2)	1.2 (1.1–1.4)	**< 0.001**
Endometrial thickness one day prior to ovulation, mm	10.8 (9.4–12.4)	10.7 (9.2–12.0)	10.2 (9.0–11.5)	9.6 (8.7–11.3)	10.6 (9.8–11.7)	0.149
LUF, n (%)	8 (47%)^d^	4 (15.4%)	6 (13.0%)	7 (9.7%)	0.0	**0.001** ^**#**^

Values are given median (25th – 75th percentiles), or n (%)

FET: Frozen embryo transfer; AUC: Area the curve; LUF: Luteinized unruptured follicle (LUF). *Independent-Samples Kruskal-Wallis Test was employed. ^#^ Chi-square test was employed

^a^ Different than 50-90p and > 90p

^b^ Different than > 90p

^c^ Different than 25–49p, 50–90p, and > 90p

^d^ Different than 10–25p, 25–49p, 50–90p, and > 90p

The comparison of the baseline demographic features and t-NC characteristics of patients with LUF or follicular collapse is presented in Table 5. Among patients with LUF, univariate comparisons revealed no significant differences in the compared characteristics, except for follicle diameter one day prior to ovulation and follicular phase peak-E_2_ levels. Specifically, when comparing patients with LUF to those with follicular collapse, the follicle diameter one day prior to ovulation was significantly higher [20.1 mm (IQR: 19.2–21.1) versus 19.2 mm (IQR: 17.8–20.9), respectively, p = 0.004], while follicular phase peak-E_2_ levels were significantly lower [284.0 pg/mL (IQR: 228.0-360.1) versus 324.5 pg/mL (IQR: 265.0-392.8), respectively, p = 0.022]. In the multivariate analysis, considering patients’ demographics and follicular phase characteristics in Table 5 within a logistic regression model, only follicular phase length (OR: 0.70, 95%CI 0.54–0.89, p = 0.004), follicle diameter one day prior to ovulation (OR: 1.4, 95%CI 1.1–1.8, p = 0.017), and follicular phase peak-E_2_ levels (OR: 0.992, 95%CI 0.986–0.998, p = 0.015) emerged as the significant independent predictors of LUF.

**Table 5:** Comparison of the baseline demographic features and true natural cycle characteristics of patients with luteinized unruptured follicle (LUF) or follicular collapse

Characteristics	LUF n = 25	Follicular collapse n = 153	*P* ^*^
Age, years	34.0 (30.5–37.50)	36.5 (32.0–40.0)	0.135
Body mass index, kg/m^2^	23.4 (22.0–26.1)	22.7 (20.9–25.8)	0.305
Antral follicle count on Day 2/3, n	16 (9.5–18.0)	14 (8–19.0)	0.539
Cause of infertility, n (%)			0.770
Unexplained infertility	6 (24.0)	51 (33.3)	
Male factor	10 (40.0)	44 (28.8)	
Advanced maternal age and/or diminished ovarian reserve	5 (20.0)	43 (28.1)	
Tubal factor	1 (4.0)	5 (3.3)	
Endometriosis	2 (8.0)	4 (2.6)	
Monogenic disorders	1 (4.0)	6 (3.9)	
Duration of infertility, months	24.0 (16.5–39.0)	28.5 (15.3–50.0)	0.889
Number of previous IVF cycles, median (minimum-maximum)	0 (0–3)	0 (0–8)	0.615
Previous childbirth, n (%)	7 (28.0)	28 (18.3)	0.280
Follicular phase length, day	11.0 (9.5–14.5)	13.0 (12.0–15.0)	0.052
Follicle diameter one day prior to ovulation, mm	20.1 (19.2–21.1)	19.2 (17.8–20.9)	**0.004**
Peak E_2_ level, pg/mL	284.0 (228.0–360.1)	324.5 (265.0–392.8)	**0.022**
Peak LH level, IU/L	39.2 (31.3–54.0)	47.7 (36.4–59.3)	0.246
Follicular phase peak P_4_ level, ng/mL	1.0 (0.9–1.4)	1.1 (0.9–1.4)	0.121
Endometrial thickness one day prior to ovulation, mm	10.9 (9.9–11.4)	10.1 (9.0–11.7)	0.084
Serum P_4_ concentration on FET-1, ng/mL	9.3 (5.5–15.4)	13.6 (10.3–17.3)	**0.002**

Values are given as median (25th – 75th percentiles), or n (%)

^*^The Mann-Whitney U test was employed to compare continuous variables, while the Chi-square test was used to compare proportions

IVF: in-vitro fertilization, FET: Frozen embryo transfer

### Multivariate analysis

**Fig. 1:**
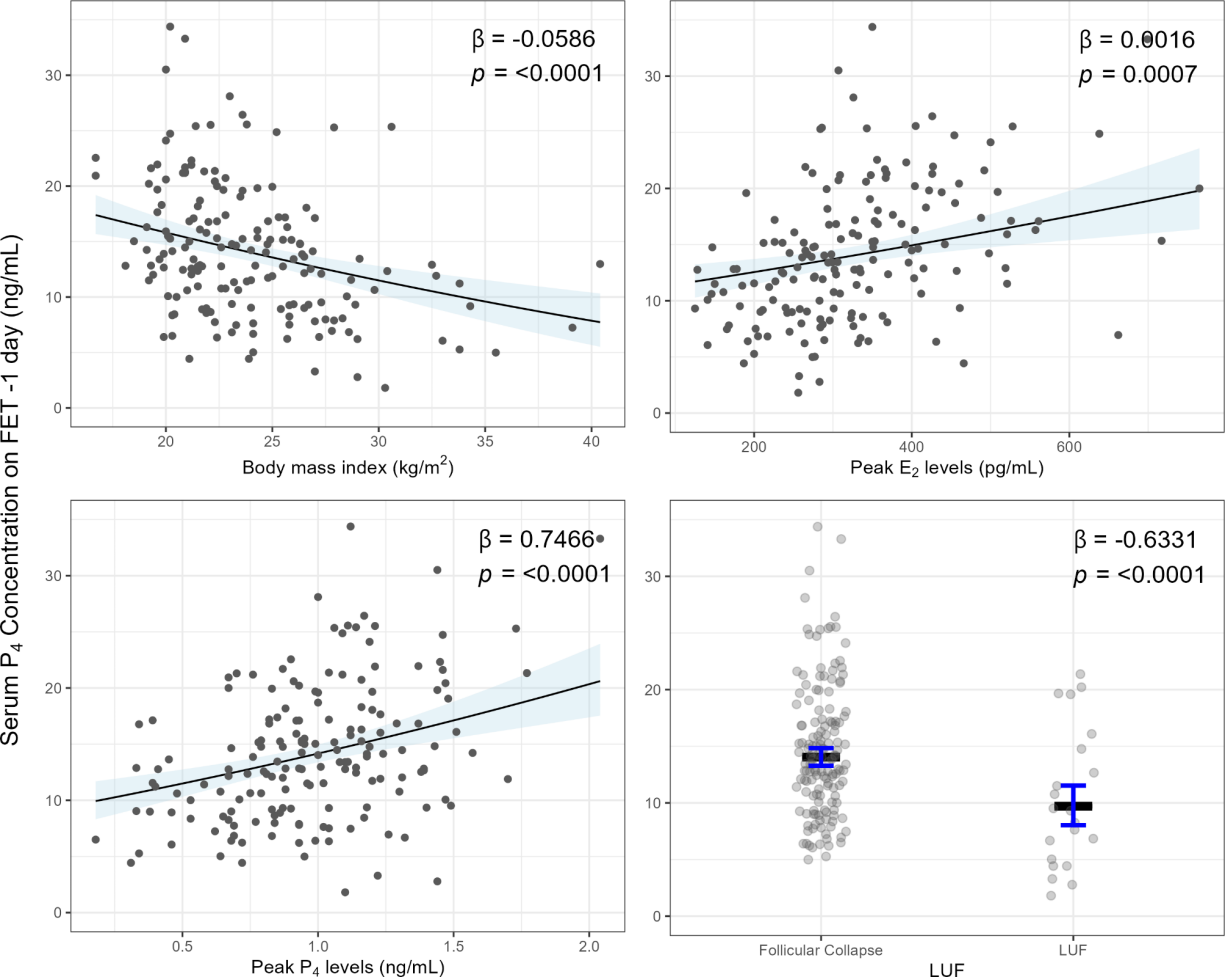
Partial effect plots of the body mass index (BMI; kg/m²), follicular phase peak-E_2_ (pg/mL), follicular phase peak-P_4_ (ng/mL), and luteinized unruptured follicle (LUF) to predict the square root of serum P_4_ levels on frozen embryo transfer (FET)-1 day (ng/mL) using the stepwise linear regression model. [Intercept: 3.89 (95% CI 3.09–4.70)]

Linear stepwise regression was performed to identify the independent predictors of serum P_4_ levels on FET-1. The covariates included in the model were age, BMI, AFC, follicular phase length, follicle diameter, and endometrial thickness one day prior to ovulation, LUF, follicular phase peak-E_2_, peak LH, and peak-P_4_ levels. Among these tested variables, BMI, LUF, follicular phase peak-E_2_, and peak-P_4_ levels were noted to be independent predictors of serum P_4_ levels on FET-1 day (Fig. 1). With this model, the square root of serum P_4_ concentration on FET-1 was noted to decrease by 0.059 for each kg/m^2^ increase in BMI (95%CI -0.084; -0.033, p < 0.001). This figure was noted to increase by 0.0016 for follicular phase peak-E_2_ level (95%CI 0.0007; 0.0025, p = 0.001), and 0.747 (95%CI 0.431; 1.062, p < 0.001) for follicular phase peak-P_4_ level. LUF was also noted to be a negative significant predictor of serum P_4_ concentration in the linear stepwise regression model (β-Coefficient: -0.633, 95%CI -0.936; -0.331, p < 0.001).

### The ROC curve analysis for predicting patients with serum P_4_ < 9.3 ng/ml (< 25th percentile) on FET-1

**Fig. 2:**
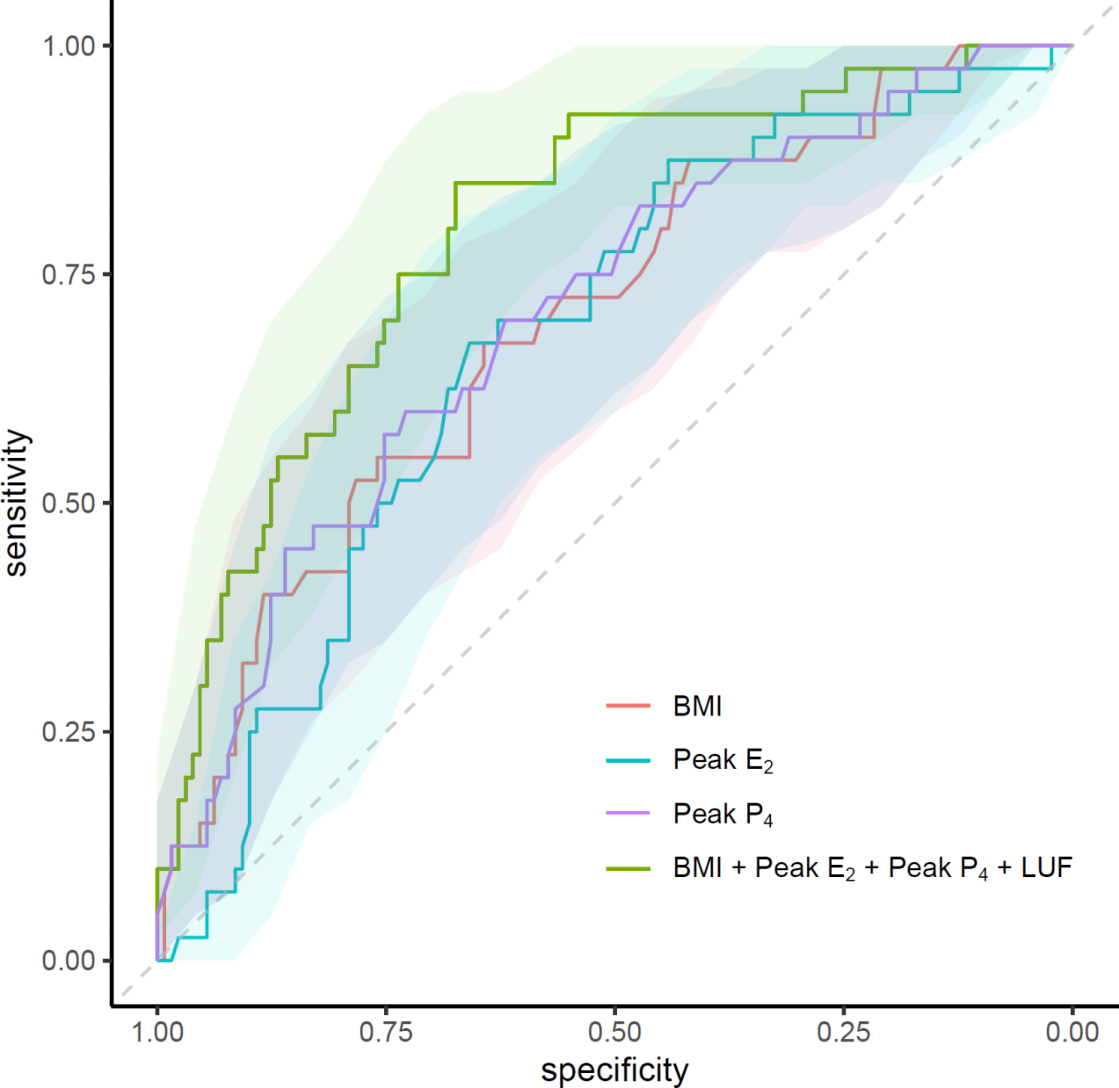
The receiver operating characteristic (ROC) curve analysis plot for body mass index (BMI; kg/m^2^) [0.70 (95%CI 0.61–0.79, p < 0.001], follicular phase peak-E_2_ (pg/mL) level [0.68 (95%CI 0.59–0.77, p < 0.001)], peak-P_4_ (ng/mL) level [0.71 (95%CI 0.61–0.80, p < 0.001)], and the combination of these three parameters plus luteinized unruptured follicle (LUF) [0.80 (95%CI 0.72–0.88, p < 0.001)] to predict low serum P_4_ levels on frozen embryo transfer (FET) -1 day (< 25th percentile; <9.3 ng/mL)

The ROC curve analysis was performed using coefficients derived from the linear stepwise regression model to evaluate the significance of each parameter on serum P_4_ levels on FET-1. The AUC for BMI was 0.70 (95%CI 0.61–0.79, p < 0.001) with a cut-off point of 23.85 kg/m^2^ (specificity of 64.3% and sensitivity of 67.5%). The AUC for follicular phase peak-E_2_ levels was 0.68 (95% CI 0.59–0.77, p < 0.001) with a cut-off point of 290.5 pg/mL (specificity of 65.9% and sensitivity of 67.5%). For follicular phase peak-P_4_ levels, the AUC was 0.71 (95% CI 0.61–0.80, p < 0.001) with a cut-off point of 0.87 ng/mL (specificity of 72.9% and sensitivity of 60.0%). Figure 2 displays the ROC curve analysis plots for BMI, follicular phase peak-E_2_, and peak-P_4_ levels. Notably, the AUC for the combination of all four independent predictors in predicting low serum P_4_ on FET-1 was 0.80 (95%CI 0.72–0.88, p < 0.001). In multivariate logistic regression analysis, the adjusted odds ratio of patients with LUF for having < 9.3 ng/ml P_4_ levels on FET-1 was found to be 4.97 (95%CI 1.66–14.94, p = 0.004) when compared to those with follicle collapse.

## Discussion

In the current study, a negative independent correlation was noted between BMI, LUF, and serum P_4_ levels on FET-1. We found a positive independent correlation between follicular phase peak-E_2_, peak-P_4_ levels, and serum P_4_ levels on FET-1. With the inclusion of these four covariates in the ROC curve analysis, the AUC for the prediction of low serum P_4_ levels on FET − 1 (< 25th percentile; <9.3 ng/ml) was ~0.80. LUF was independently associated with a ~five-fold increase in the odds of having <9.3 ng/ml P_4_ levels on FET-1.

Timely and optimal exposure of the endometrium to progesterone is crucial for the establishment and maintenance of an ongoing pregnancy. The presence of ovulation in regularly cycling women does not secure a receptive endometrium in all cycles [10]. In regularly cycling women, a suboptimal preovulatory follicular development alongside low late-follicular/mid-cycle hormone profiles may result in a suboptimal luteal P_4_ profile and endometrial milieu [19, 20]. Unfortunately, there is a paucity of data on the correlation between follicular phase endocrine and ultrasonographic parameters and mid-luteal P_4_ levels [19, 21–23] and reproductive outcomes in spontaneous [24–27] and NC FET cycles [28–31].

Despite the paucity of data, it is generally assumed that an optimal luteal function in NC requires optimal pre-ovulatory follicular development and steroidogenesis [32, 33]. Soules et al. [21] studied factors controlling corpus luteum function in 14 volunteers during a spontaneous cycle. Although there was a significant positive correlation between the mean follicle diameter and serum AUC-E_2_ during the late follicular phase, these parameters did not correlate with P_4_ production during the luteal phase [21]. However, a significant association between late follicular phase E_2_ and mid-luteal P_4_ was reported by another prospective analysis of 192 regularly cycling women [22]. In the current study, we noted a positive correlation between follicular phase peak-E_2_ and peak-P_4_ levels, and serum P_4_ levels on FET-1.

An estrogen-induced proliferative endometrium before P_4_ exposure is a prerequisite for a receptive endometrium in an NC [34]. Regarding the impact of follicular E_2_ levels on reproductive outcomes, significantly higher salivary mid-follicular E_2_ levels [25], urinary [27], and serum [35] periovulatory E_2_ levels have been reported in spontaneous conception cycles when compared to non-conception cycles. Romanski et al. reported that women with elevated E_2_ levels (> 100 pg/mL) until the LH surge for > 4 days had higher LBRs when compared to those with ≤ 4-days duration after warmed blastocyst transfer in a t-NC [30]. The authors concluded that the duration of elevated E_2_ levels, rather than the amplitude, during the late follicular phase, may be a predictor of a receptive endometrium in the t-NC FET [30]. In the current study, we noted a positive correlation between follicular phase peak-E_2_ levels and serum P_4_ concentration on FET-1.

In theory, differences in the amplitude and duration of the LH surge might result in differences in the AUC for LH as the driving force of P_4_ production by the corpus luteum and, hence, may have implications for the reproductive outcome in t-NC FET [36]. However, Soules et al. reported no correlation between the AUC-LH surge and the luteal P_4_ secretion [21]. Although the mid-luteal serum P_4_ levels were lacking, peak-LH levels [26], and the duration of the LH surge [24] have been reported to be associated with reproductive outcomes in spontaneous cycles. In the current study, neither the AUC-LH nor the peak-LH levels were noted to be the significant predictors of serum P_4_ levels on FET-1.

Following the LH surge in NC, resumption of meiosis occurs at low LH levels, whereas adequate luteinization requires higher LH levels [37]. In contrast, follicle rupture is only achieved at very high LH levels [37]. In the rat model, the threshold LH level required for resumption of meiosis and P_4_ secretion is only 5% of the peak level, whereas the threshold is > 85% of the peak level for follicular rupture [38]. The hierarchic level-response effect of LH explains LUF with the lack of follicle wall rupture with blunted LH surges, despite luteinization and hence serum P_4_ rise [19, 39–41]. Moreover, LUF cycles are typically characterized by luteal phases of normal duration; however, with lower mid-luteal serum P_4_ levels in spontaneous cycles [39–42] and NC FET [23]. In line with these previous studies, among the patients in the lowest (< 10th percentile) category of serum P_4_ on FET-1, 8 out of 17 cycles (47%) were characterized as LUF cycles, with serum P_4_ levels ranging from 1.8 to 6.8 ng/mL. Moreover, LUF was noted to be a significant independent predictor for low luteal serum P_4_ levels on FET-1.

In patients with LUF, aside from the significantly higher follicle diameter one day prior to ovulation and the significantly lower follicular phase peak-E_2_ levels compared to patients with follicular collapse, all the other demographic and the t-NC characteristics were comparable. Despite the limited sample size for such a comparison, in logistic regression analysis, shorter follicular phase length, a higher follicle diameter one day prior to ovulation, and lower follicular phase peak-E_2_ levels were identified as independent predictors of LUF.

Although not within the scope of the current study, conflicting data exist on the impact of LUF on reproductive outcomes in t-NC, some reporting a detrimental effect [43], whereas, others reporting no effect [23, 44]. Our findings suggest that LUF carries a risk of suboptimal serum P_4_ levels on FET-1 (adjusted-OR: 4.97, 95% CI 1.66–14.94) and hence, may be a risk factor for suboptimal reproductive outcomes following t-NC FET. Therefore, recognition of LUF may permit the identification of those cases that may need exogenous progesterone administration for LPS in t-NC FET. Alternatively, a routine policy of LPS in all t-NC FET cycles may alleviate such cases with suboptimal P_4_ levels without necessitating the recognition of LUF. The need for frequent visits to recognize LUF and the increased financial burden associated with routine LPS are the drawbacks of these two different policies.

After applying stepwise elimination in the linear regression model, we noted that BMI was one of the significant independent predictors of serum P_4_ levels on FET-1. For the prediction of low serum P_4_ levels on FET-1, in the adjusted ROC curve plot analysis, the AUC for BMI was 0.70 (95%CI 0.61–0.79, p < 0.001) with a cut-off point of 23.85 kg/m^2^. In line with the current study, a negative correlation between mid-luteal serum P_4_ levels and BMI was also reported in spontaneous [45] and t-NC FET cycles [11].

Two studies previously explored the impact of mid-luteal serum P_4_ levels on reproductive outcomes in t-NC FET [11, 15]. In a retrospective cohort of 294 cycles, mean serum P_4_ levels on FET-1 were significantly higher in patients who had a live birth compared to those who did not. Women with low P_4_ levels (< 10 ng/mL) had significantly lower LBRs compared to those with P_4_ levels > 10 ng/mL (25.7% versus 41.1%) [11]. A recent RCT evaluating the role of routine LPS in t-NC FET (on days 2, 3, and 5) noted that the LBR increased by ~10% by LPS; however, mean serum P_4_ levels on the day of FET were not associated with LBR in the two groups receiving LPS or not [15]. In the group with no LPS, patients with low serum P_4_ levels (< 29 nmol/L) on the day of FET had comparable LBRs when compared to their counterparts with serum P_4_ levels >29 nmol/L [15]. The inclusion of cleavage and blastocyst stage transfers and measurement of P_4_ measurement on different days and timings (on days 2, 3, and 5) are important limitations of that study [15]. In the era of “personalized treatment,“ identification of women with low serum P_4_ on FET-1 (e.g., high BMI, those with LUF, low follicular phase peak-E_2_, and peak-P_4_ levels) would permit the administration of LPS in selected cases, only instead of a routine LPS for all t-NC FET.

The strength of the current study is the inclusion of consecutive 178 ovulatory patients with serial endocrine and ultrasonographic monitoring in all patients. Moreover, to our knowledge, the current study is the first to explore the association between patient, follicular phase characteristics, and luteal function in warmed blastocyst transfer cycles employing t-NC. Although the retrospective design and single-point of assessment serum P_4_ on FET-1 are limitations, serum P_4_ concentrations were prospectively monitored one day prior to warmed blastocyst transfer at strict time points during 12.00–1.00 pm.

In conclusion, BMI, LUF, peak-E_2,_ and peak-P_4_ levels are independent predictors of low serum P_4_ levels on FET-1 (< 25th percentile; <9.3 ng/ml) in t-NC FET cycles. Recognition of risk factors for low serum P_4_ on FET-1 may permit a personalized approach for LPS in t-NC FET to maximize reproductive outcomes.

## Data Availability

Data will be made available on request.
